# NAA at a high concentration promotes efficient plant regeneration *via* direct somatic embryogenesis and SE-mediated transformation system in *Ranunculus sceleratus*

**DOI:** 10.1038/s41598-019-54538-8

**Published:** 2019-12-04

**Authors:** Ke-dong Xu, Wei Wang, De-shui Yu, Xiao-li Li, Jia-min Chen, Bo-jin Feng, Ya-wen Zhao, Meng-jia Cheng, Xin-xin Liu, Cheng-wei Li

**Affiliations:** 1 Key Laboratory of Plant Genetics and Molecular Breeding, Zhoukou Normal University, Zhoukou, 466001 China; 2 College of Life Science and Technology, Henan Institute of Science and Technology, Xinxiang, 453003 China; 3 Henan Key Laboratory of Crop Molecular Breeding and Bioreactor, Zhoukou Normal University, Zhoukou, 466001 China

**Keywords:** Organogenesis, Plant embryogenesis

## Abstract

The novel methods for efficient plant regeneration *via* direct somatic embryogenesis (SE) and SE-mediated transformation system under high concentration of NAA in *Ranunculus sceleratus* were established. On MS media containing a high concentration of NAA (10.0 mg/L) in the dark, all inoculated explants (root, stem and leaf) formed somatic embryos at high frequencies, respectively, 66.03, 126.47 and 213.63 embryoids per explant, and 100% of the embryoids developed into plantlets on 1/2 MS rooting media. Morphological and histological analyses revealed that SE in *R. sceleratus* followed a classical pattern. All inoculated explants can be used as receptors for genetic transformation in *R. sceleratus*, through direct SE-mediated method after *Agrobacterium* infection. *RcLEC1-B*, as a marker gene, changed the number and morphology of flower organs and the development of cuticle in *R. sceleratus*, which indicated that the efficient transgenic system of *R. sceleratus* was established. To our knowledge, this is the first observation that both direct SE and transgenic transformation system, *via* induction of a single plant growth regulator, have been successfully constructed in *R. sceleratus*.

## Introduction

There are a variety of medicinal plants and ornamental flowers in Ranunculaceae family, especially the diploid plants, such as *Aquilegia coerulea* and *Nigella damascene*, which are ideal recipient system for the molecular mechanism research of flower development. *Ranunculus sceleratus* is a high rich source of valuable secondary metabolites, including alkaloids, tannins, flavonoids, isoscopoletin and protocatechuyl aldehyde^1^. It has been used as a traditional medicine in China for a long history^2^. *R. sceleratus* was always employed to prevent the replications of HBV (Hepatitis B virus) and HSV-1 (Herpes simplex virus type-1)^1^, and in the treatments of jaundice, rheumatic pains, asthma and urinary incontinence^3^. *R. sceleratus* is very effective as an insecticide against *Drosophila melanogaster* and *Tribolium castaneum*^4^. In addition, *R*. *sceleratus* has a high capacity for sewage disposal, it can absorb large amounts of nitrogen and phosphorus, accumulate and monitor the variation of heavy metals, such as Cu, Pb, Fe and Zn^2,5^, etc. However, until now, it has no a universal and stable genetic transformation method used in Ranunculaceae family, which greatly limits the research on the basic research and application research of Ranunculaceae plants.

In the process of plant somatic embryogenesis (SE), the induced somatic cells promote proliferation and dedifferentiation under an appropriate stimulus, for instance, plant growth regulators (PGRs), stress factors, spontaneous factors, etc., then initiate embryo development through redifferentiation^6,7^. Somatic embryogenesis structures can be used as recipient explant for genetic transformation systems^8^, which can generate transgenic plants with potential as industrial sources of useful compounds and thus act as plant bioreactors^9^; for example the hairy roots of *Silene vulgaris*^10^ and secondary metabolite production^11^. However, until now, the efficient regeneration and transgenic system of Ranunculaceae family has not been established; there has been no report of direct SE induction of *R*. *sceleratus*, although previous landmark studies have been done on Ranunculus sp., such as *R. asiaticus*^12,13^. Here, we present a regeneration system *via* direct SE and SE-mediated transgenic system of *R*. *sceleratus*, which will contribute to the variety improvement, the construction of SE based bioreactor, and the basic theoretical research of Ranunculaceae plants as a reference system.

## Materials and Methods

### Plant materials and explant preparation

Seeds of *R. sceleratus* were generously provided by Prof. Le Zhao (College of Pharmacy, Henan University of Chinese Medicine, Henan, China), which were sterilized with 75% (v/v) ethanol for 0.5–1 min, rinsed 3–5 times with sterilized distilled water, then soaked in 2.5% (v/v) sodium hypochlorite for 9–10 min and rinsed 3–5 times with sterilized distilled water. The sterilized seeds were placed on MS medium supplemented with 30 mg/L sucrose and 8.0 g/L agar (pH 6.06) to obtain sterile seedlings; an average of 15 to 20 seeds per bottle was used. The germinating seeds were cultivated at 25 °C with a 16 h light/8 h dark photoperiod (150–180 µmol·m^−2^s^−1^). After two weeks of cultivation, the seedling height is about 4–5 cm and developed into plantlets with roots, which is the optimal state for induction of SE.

### Induction of SE

Leaf discs about 1–1.5 cm^2^ in area, and root and stem segments about 1–2 cm long (without axillary buds on stem segments) were excised from *R. sceleratus* plantlets for use as explants for the induction of somatic embryogenesis. The explants were put on MS media with 30 g/L sucrose and 3.6 g/L gellan gum (pH 5.8) supplemented with 1-naphthaleneacetic acid (NAA) at concentrations of 1.0, 2.5, 5.0, 10.0 and 20.0 mg/L. The inoculated explants were cultivated at 25 ± 1 °C in the dark or under low light (60 µmol·m^−2^s^−1^) conditions to induce SE. The process of SE development was recorded using a digital camera (EOS 600D, Canon Inc., Japan) and a stereomicroscope (SMZ800, Nikon Corporation, Japan). To evaluate the frequency of SE from explants, thirty replicates of each of 10 explants were used.

### Histological analyses of SE

Frozen sections of somatic embryos at different developmental stages were prepared for microscopy according to a previously published method^13^, and imaged using an optical microscope (BX 41, Olympus Corporation, Japan).

### Plantlet formation from somatic embryos

Without no change in the media, the PGR supplement and the dark conditions, somatic embryos spontaneously developed into plantlets. When plantlets had reached a length of 1–2 cm they were separated and transferred to rooting medium (1/2 MS salt + 1/2 B5 vitamins + 10 g/L sucrose + 8.0 g/L agar, pH 5.8) to induce root formation. To evaluate the frequency of regeneration of plantlets from somatic embryos, thirty replicates of each of 10 somatic embryos were set.

### Isolation and overexpression vector construction of *RcLEC1-B*

The RNA isolation, DNase treatment, reverse transcription and isolation of *RcLEC1-B* (*LEAFY COTYLEDON1-B*) were made according to the published method^14^. The nucleotide sequence was submitted to GenBank, was deposited in GenBank under accession number KM115581.1. The *RcLEC1-B* ORF was cloned into the *Kpn*I and *Bam*HI sites of pCAMBIA2300 to produce the overexpression vector pCAMBIA2300-*RcLEC1-B*. This vector was introduced into *Agrobacterium tumefaciens* (GV3101) by means of freeze-thawing^14^. In addition, the binary expression vector pBI121 carrying *GUS* reporter gene was used to analysis of transgenic efficiency *via* GUS histochemical assay.

### Plant transformation *via* direct SE

The root, stem and leaf explants were immersed in *Agrobacterium* suspension (1/2 MS salt + 1/2 B5 vitamins + 50 g/L sucrose, pH 5.8) and uniformly shocked in the dark for 8–10 min. The explants were dried with sterile tissue paper and transferred to co-culture medium (MS salt + B5 vitamins + 40 mg/L acetosyringone (AS) + 30 g/L sucrose + 8.0 g/L agar, pH 5.8) and kept in the dark for 72 h. Then the explants were placed on the selective culture medium, with composition MS salt + B5 vitamins + 10 mg/L NAA + 100 mg/L Kan + 500 mg/L Carb + 30 g/L sucrose + 3.6 g/L gellan gum (pH 5.8). Formed embryoids on explants were isolated and transferred to rooting medium.

### GUS histochemical assay on somatic embryos

*GUS* histochemical assay was performed with Kan-resistant positive embryoids according to Jefferson’s protocol^15^ with some modification. The embryoids were stained with 0.96 mM 5-bromo-4-chloro-3-indolyl β-D-glucuronide (X-Gluc) (dissolved by N, N-dimethylformamide) in PBS (0.1 M, pH 7.0) containing 0.5 mM EDTA, 5 mM potassium ferricyanide, 5 mM potassium ferrocyanide, 2% carbinol and 0.1% (v/v) Triton X-100, overnight under 37 °C. The chlorophyll was removed by using 100% ethanol after X-Gluc staining. Then samples were rehydrated in ethanol series 75, 37.5 and 18.75% ethanol (15 min each step), placed in 5% ethanol and 25% glycerol for 30 min, preserved in 50% glycerol for further observation and photograph.

### Statistical analysis

Using SPSS 16.0 software, analyses of variance (ANOVA) with 99% and 95% confidence intervals were applied to the digital data.

## Results

### Induction of SE from root, stem and leaf explants on media with NAA in the dark

**Figure 1:**
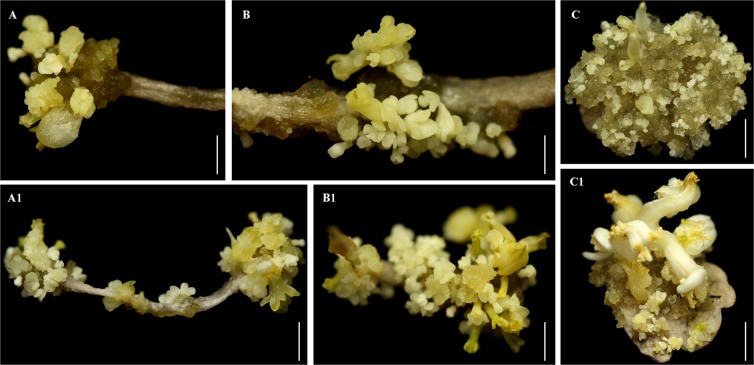
Induction of somatic embryogenesis from root, stem and leaf explants of *R. sceleratus*. (**A**–**C**) Early developmental stages; (**A1**–**C1**) Late developmental stage; (**A**,**A1**) Root explants; (**B**,**B1**) Stem explants; (**C**,**C1**) Leaf explants. Bars (**A**,**C**,**A1**,**B1**,**C1**) = 0.5 cm; Bar (**B**) = 0.25 cm.

**Figure 2:**
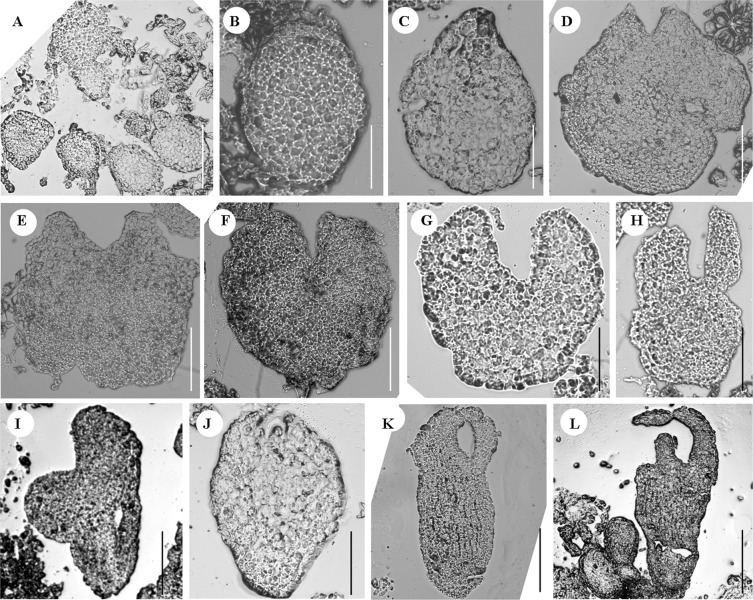
Microscopic images of different developmental stages of somatic embryogenesis in *R. sceleratus*, obtained using frozen section technique. (**A**) Multiple embryoids induced from leaf explant; (**B**) Globular embryo; (**C**) Transitional globular-heart embryo; (**D**–**G**) Heart embryos; (**H**) Torpedo-shaped embryo; (**I**–**L**) Cotyledon embryos. Bar (**A**) = 100 μm; Bars (**B**,**C**) = 50 μm; Bars (**D**–**G**) = 75 μm; Bar (**H**) = 200 μm; Bars (**I**,**J**) = 200 μm; Bars (**K**,**I**) = 400 μm.

To establish the SE induction system, NAA at concentrations of 1.0, 2.5, 5.0, 10.0 and 20.0 mg/L was tested in order to optimize PGR conditions, and different types of explants from root, stem and leaf material were investigated. The results showed that with all the investigated supplementary concentrations of NAA in the media, SE was successfully induced from all of the explant types tested, though at different induction frequencies. However, no SE induction was observed from root, stem or leaf explants on media without NAA (Table 1). Of the concentrations tested, 1.0 mg/L NAA resulted in the smallest average numbers of induced somatic embryos (9.33 from leaf, 6.57 from stem and 3.90 from root) and only a small amount of rhizoids^16^ per explant (Table 1). However, a concentration of 2.5 mg/L NAA also resulted in a relatively small amount of SE and produced a few frog egg-like bodies (FELBs)^17^ per explant (Table 1). A concentration of 10.0 mg/L NAA resulted in the highest average numbers of somatic embryos (66.03 from leaf, 126.47 from stem and 213.63 from root) induced per explant (Table 1). These results showed that for *R. sceleratus* leaf explants are the best type to use (Fig. 1), and that 10.0 mg/L NAA is the optimal PGR concentration. We also tested the effect of light; this experiment showed that under light conditions no SE was induced from any of the types of explant tested on media containing any of the above-mentioned concentrations of NAA, suggesting that incubation under dark conditions is necessary for SE induction in *R. sceleratus*. We found that the embryoids induced exhibited the five classic developmental stages in terms of morphological structure: multicellular pro-embryo, globular embryo, heart-shaped embryo, torpedo-shaped embryo, and cotyledon embryo (Fig. 2). However, there were many kinds of cotyledon embryos with different numbers of cotyledons, with two, three, four and multiple cotyledons all being observed in cotyledon embryos (Fig. 1A,B,A1,B1).

**Table 1:** Effect of NAA at different concentrations on induction of somatic embryos from root, stem and leaf explants of *R. sceleratus*.

NAA (mg/L)	Number of somatic embryos induced per explant		
	Root	Stem	Leaf
0	0.00 ± 0.00 kJ	0.00 ± 0.00 kJ	0.00 ± 0.00 kJ
1.0	3.90 ± 0.44 jkIJ	6.57 ± 0.42 ijHI	9.33 ± 0.49 iH
2.5	14.80 ± 0.44 hG	20.03 ± 0.73 gF	19.37 ± 0.74 gFG
5.0	22.60 ± 0.55 gEF	27.47 ± 0.62 fE	35.17 ± 0.68 eD
10.0	66.03 ± 0.80 cC	126.47 ± 2.23 bB	213.63 ± 4.76 aA
20.0	27.33 ± 0.85 fE	35.13 ± 0.60 eD	39.23 ± 0.50 dD

The mean and standard error per treatment were calculated from 300 explants on 30 petri dishes (treated as 30 replicates). Capital and lowercase letters indicate a significant difference at the 1% and 5% probability level respectively. Significant differences were analyzed with a Duncan test using SPSS 16.0.

### Frozen sectioning revealed the development process of somatic embryos

A frozen sectioning technique was employed to analyze the developmental process during SE. As SE progressed, globular embryo (Fig. 2B), heart-torpedo embryo (Fig. 2D–G), torpedo-shaped embryo (Fig. 2H), and cotyledon embryo (Fig. 2I–L) were formed.

### Plantlet formation from somatic embryos *in vivo* and GUS histochemical assay

**Figure 3:**
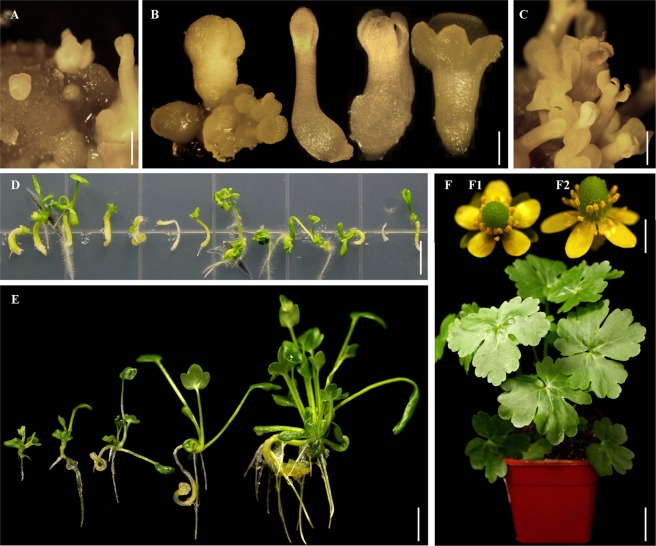
The process of regeneration of *R. sceleratus via* SE. (**A**) Multiple embryoids at different developmental stages induced on leaf explant; (**B**) The embryoid developmental process, showing many kinds of cotyledon embryos with different numbers of cotyledons; (**C**) Mature embryoids; (**D**) The induction of roots from embryoids; (**E**) Regenerated plantlets; (**F**) A regenerated plant; (**F1**,**F2**) The flowers of regenerated plant. Bar (**A**) = 200 μm; Bar (**B**) = 300 μm; Bar (**C**) = 400 μm; Bar (**D**) = 0.5 cm; Bar (**E**) = 1 cm; Bar (**F**) = 2 cm; Bars (**F1**,**F2**) = 0.35 cm.

**Figure 4:**
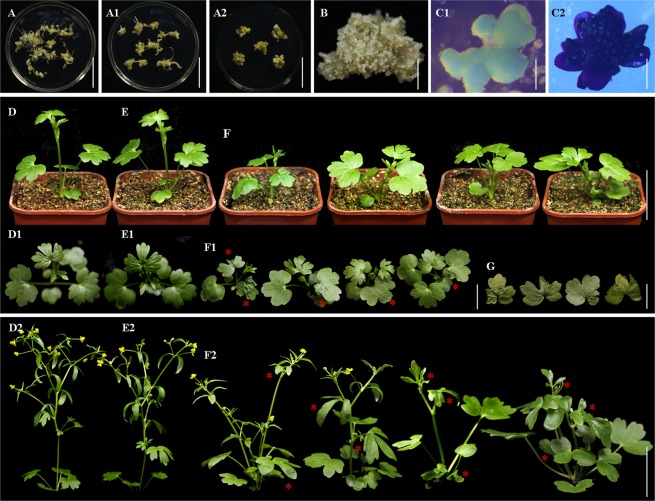
Overexpression of *RcLEC1-B* results in dwarfing and abnormal flower organs in *R. sceleratus*. (**A**,**A1**,**A2**,**B**) The transformation process of *R. sceleratus*. Multiple kan-resistant positive embryoids at different developmental stages induced on root, stem and leaf explants; (**A**) Root explant; (**A1**) Stem explant; (**A2**,**B**) Leaf explants; (**C1**) GUS assay on positive embryoids; (**C2**) GUS assay on positive flower; (**D**,**D1**) Wild-type plants (15 d); (**E**,**E1**) Empty vector (pCAMBIA2300) plant (15 d); (**D2**) Wild-type plant (30 d); (**E2**) Empty vector plant (30 d); (**F**,**F1**) *RcLEC1-B*-OE *R. sceleratus* with dwarfing plant-type (15 d); (**F2**) *RcLEC1-B*-OE plants (30 d); (**G**) The cuticle defects on leaves of *RcLEC1-B*-OE *R. sceleratus*. Bar (**A**,**A1**,**A2**) = 3 cm; Bar (**B**) = 1 cm; Bar (**C1**) = 500 μm; Bar (**C2**) = 5 mm; Bars (**D**,**D2**,**E**,**E2**,**F**,**F2**) = 3.5 cm; Bars (**D1**,**E1**,**F1**) = 1 cm; Bar (**G**) = 1 cm.

Plantlets could be induced *in vivo* from *R. sceleratus* somatic embryos. Somatic embryos produced *in vivo* on the induction medium was able to spontaneously develop into plantlets; each somatic embryo structure generally developed only one plantlet (Fig. 3B,D,E). The formed somatic embryos were stained in blue after GUS-staining (Fig. 4C1).

### Overexpression of *RcLEC1-B* results in dwarfing and abnormal flower organs

**Figure 5:**
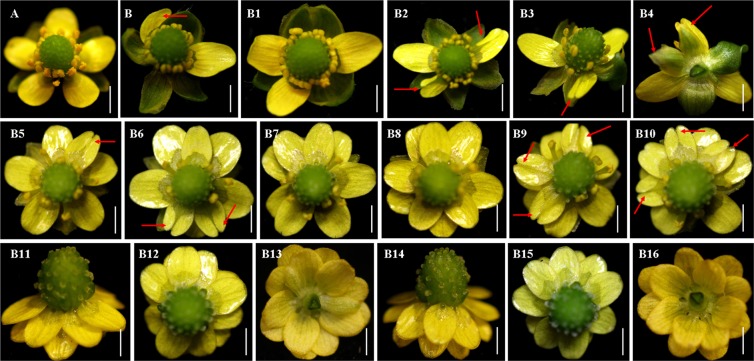
Morphology and numbers of floral organs of wild-type plant and *RcLEC1-B*-OE *R. sceleratus*. (**A**) Wild-type plant with five sepals, five petals, sixteen stamens (always more than ten) and one aggregate pistil (always including almost 100 achenes); (**B–B16**) *RcLEC1-B*-OE flowers; (**B**–**B10**) *RcLEC1-B*-OE flowers contained unnormal transition petal structures; (**B4**) Indistinguishable structures intermediate between sepals and petals; (**B11**–**B16**) No sepal and stamen. The red arrows mark the developmental malformed petals. Bars (**A**,**B–B16**) = 2 mm.

Overexpression of *RcLEC1-B* in *R. sceleratus* resulted in slightly dwarfed plants with folded leaves (Fig. 4F,F1,F2,G) for the defective cuticle. The abnormal androecium and gynoecium were observed in *RcLEC1-B*-OE *R. sceleratus*. *RcLEC1-B*-OE affected the variable numbers in floral organs per flower in *R. sceleratus* with 0–5 sepals, 2–14 petals and 0–16 stamens; especially, the transition state petals with obvious notch appearance in *RcLEC1-B R. sceleratus* (Fig. 5B,B2–B6,B9,B10). In the statistics of the efficiency of genetic transformation, we found that the highest transformation efficiency was approximately 70%, was obtained in leaf explant; that of the stem explant was 40%, and the minimum transformation efficiency was only 20% in root explant.

## Discussion

Somatic embryogenesis is model system for reveal the developmental mechanism of plant zygotic embryo and a valuable tool for plant regeneration^6,18^. The results of previous studies showed that various treatments could be successfully applied to the induction of SE in higher plants; these conditions include PGRs, such as 2,4-D^17,19^, NAA^16^, TDZ^16,19^, and IAA^20^; desiccation^20^; osmotic stress^21^; polyamines^22^, sugars^23–26^, salts^27^, and metal ions^28^. Always, the PGRs with suitable concentration, variety and combination are very important to SE and plant propagation^29^. In our study, it showed that the induced effect of SE under high concentration NAA (10.0 mg/L) is optimal, however, it needs to be strictly dark. The induction of SE in *R. sceleratus* under light was failure, which was consistent with the results of FELB in *Solanum nigrum*^17^. On the contrary, the induction of PLB structure in *Rosa canina* need involve high light condition^19^, and in *Trichosanthes kirilowii* the second stage of induction of rhizoid tubers (RTB), after rhizoid induction, also required light^16^. Optimization of light conditions is therefore important for the induction of different somatic embryo structures in different plant species. In this study, we found that the optimum concentration of NAA on induction of SE in *R. sceleratus* was 10.0 mg/L, which was much higher than that (1.0 mg/L) on induction of RTB in *T*. *kirilowii*^16^. The same PGR with different concentrations always can successfully induce different SE structures, which indicates that the concentration of PGR plays a key role in plant SE activation, however, the specific mechanism is still unclear. In this study, all types of explants have the potential to successfully induce SE, nevertheless, the induction results indicated that the leaf was the best explant with a higher frequency on SE induction compared with stem and root explants.

We conclude that the SE induction of *R*. *sceleratus* in this report, possess some traits based on the following reasons. In summary: (1) To our knowledge, this is first report of a method for the direct induction of SE in *R. sceleratus*; (2) There are five classic stages in the development of somatic embryo structures: pro-embryo, heart-shaped embryo, globular embryo, torpedo-shaped embryo and cotyledon embryo (Fig. 2). (3) We observed many kinds of cotyledon embryos with cotyledon numbers ranging from two to four, and also multiple cotyledons on polymeric cotyledon embryos (Figs. 1 and 3); (4) The results showed that SE often form individually on the surface of explants; (5) Darkness is necessary for induction; (6) The regeneration system *via* SE has a high regeneration efficiency (66.03–213.63 plantlets per explant), and it is therefore reasonable to predict that *R. sceleratus* transformation based on this regeneration system might also be high-efficiency. Further study is needed to determine whether the induction pathway developed here for *R. sceleratus* can be established in other species of Ranunculaceae.

The transcription factor *LEC1-B*^14^, one of the CCAAT box-binding factors (CBFs) known as the *HAP3* (*HEME-ACTIVATED PROTEIN3*) group, was isolated in protocorm-like body (PLB)^19^, a special SE structure of *Rosa canina*. It was proved in our previous studies that *RcLEC1-B*-OE significantly changed the number and morphology of floral organs, formed the transition state structures and regulated the development of cuticle in Arabidopsis^14^. In this study, the same results were obtained in *RcLEC1-B*-OE *R. sceleratus*, which changed in the numbers of reproductive organs, absence of floral organs, especially, the transition state structures between petal and sepal. The above results confirmed that the same function of *RcLEC1-B* gene was verified in *R. sceleratus*, and the genetic transformation system of *R. sceleratus via* SE has been successfully constructed, together with the overexpression results of reporter gene (*GUS*). To our knowledge, this is the first observation of transgenic system *via* direct SE-mediated method, have been successfully constructed in Ranunculaceae plant. Our findings will greatly promote the researches on metabolic mechanism of secondary metabolites, molecular mechanism of heavy metals accumulation and tolerance, molecular breeding and basic research of the rare flowers of Ranunculaceae family, such as *R. japonicus*, *R. asiaticus*, *Aquilegia coerulea* and *Nigella damascene*, etc.

## Conclusion

Here, we applied SE approach to established efficient plant regeneration and transformation system in *R. sceleratus*. The high concentration of NAA (10.0 mg/L) is a crucial regulatory factor for the SE initiation of *R. sceleratus*. Leaf is the optimal explant formed somatic embryos at high frequencies, comparing with root and stem. All the three kinds of inoculated explants can be used as recipient materials for genetic transformation, in spite of the efficiencies of both regeneration *via* SE and transformation of leaf are higher. The SE of *R. sceleratus* followed a classical pattern indicated by morphological and histological analyses. The uniform results of *RcLEC1-B* overexpression in either *R. sceleratus* or Arabidopsis, together with GUS staining result, showed that both direct SE and transgenic transformation systems have been successfully constructed in *R. sceleratus*.
